# Phytoaccumulation of trace elements (As, Cd, Co, Cu, Pb, Zn) by *Nicotiana glauca* and *Euphorbia segetalis* growing in a Technosol developed on legacy mine wastes (Domingo Rubio wetland, SW Spain)

**DOI:** 10.1007/s10653-023-01523-w

**Published:** 2023-03-16

**Authors:** C. Barba-Brioso, P. J. Hidalgo, S. Fernández-Landero, I. Giráldez, J. C. Fernández-Caliani

**Affiliations:** 1 Department of Crystallography, Mineralogy and Agricultural Chemistry, University of Seville, Campus Reina Mercedes, s/n., 41071 Seville, Spain; 2 Department of Integrated Sciences, University of Huelva, Campus El Carmen, s/n., 21071 Huelva, Spain; 3 Department of Earth Sciences, University of Huelva, Campus El Carmen, s/n., 21071 Huelva, Spain; 4 Department of Chemistry, University of Huelva, Campus El Carmen, s/n., 21071 Huelva, Spain

**Keywords:** Soil pollution, Heavy metals, Rhizosphere, Phytoextraction, Nicotiana, Euphorbia

## Abstract

Sulfidic mine wastes have the potential to generate acid mine drainage (AMD) and release acid leachates containing high levels of iron, sulfate and potentially toxic elements (PTEs). Soils receiving AMD discharges are generally devoid of vegetation. Only a few metal-tolerant plant species can survive under such adverse soil conditions. This work investigates two plant species, *Nicotiana glauca* and *Euphorbia segetalis,* that have successfully colonized an AMD-impacted wetland area in south-western Spain. The uptake of PTEs from the soil by roots and their transfer and accumulation in the above-ground biomass were quantified. Results showed that these pioneer plants grew in patches of neutral soil within the wasteland despite the high concentrations of PTEs in the rhizosphere soil (up to: 613 mg kg^−1^ As, 18.7 mg kg^−1^ Cd, 6370 mg kg^−1^ Cu, 2210 mg kg^−1^ Pb and 5250 mg kg^−1^ Zn). The target organs of As, Cu and Pb accumulation were: root > leaf > stem in *N. glauca*, and root > stem > leaf in *E. segetalis*. Zinc and Cd showed a significant decrease in roots relative to aerial parts of *N. glauca*, and Co was preferentially partitioned in stems of *N. glauca* and leaves of *E. segetalis*. The soil–plant transfer coefficient values of PTEs in all parts of both plants were well below unity with the only exception of Cd in leaves of *N. glauca* (1.254), suggesting that roots acted as a barrier limiting the uptake of PTEs by plants. Interestingly, under the same soil conditions, *N. glauca* absorbed Cd in considerable proportions from soil and accumulated it in its leaves, while *E. segetalis* was not effective in transferring PTEs from roots shoots except for Co. In conclusion, soil pH and plant-related factors greatly influence the stabilization of PTE in the rhizospheric soil and produce inconsistencies in PTE phytoavailability. The findings of this study provide criteria to assist in natural remediation in other legacy contaminated sites worldwide.

**Supplementary Information:**

The online version contains supplementary material available at 10.1007/s10653-023-01523-w.

## Introduction

Multiple pollution sources are often found around the mouth of rivers and estuaries because population and industry tend to be clustered in distinct geographic areas with favorable natural conditions and resources, such as fertile soils, water availability, and low lands for urban development (Santucci et al., 2017). Many of these conditions converge in coastal wetland areas (Li et al., 2014), where the environmental functions and ecosystem services of the soil may be dramatically disrupted by man-induced reactions involving the formation of acid sulfate soils, oxidation of organic matter and subsequent emission of carbon dioxide to the atmosphere, and potentially toxic trace elements (PTEs) release into the nearby water courses (Fernández et al., 2010). In such degraded wetlands, soils lose their filtering and storing capacity for pollutants that are eventually discharged in high amounts from liquid industrial and agricultural effluents, solid waste deposited on illegal dumping sites, or dust deposition (Conesa et al., 2011; Grantcharova & Fernández-Caliani, 2022).

Fortunately, the economic development of countries is commonly accompanied by increased environmental awareness and protection, and so research, legislative and policy efforts are being undertaken to achieve a sustainable land use and management. Thus, for example, the European Commission (2021) has launched a new soil strategy for 2030 to ensure a high level of environmental and health protection. However, for legacy contaminated sites a common approach is lacking in the European Union, which is a relevant legal gap concern.

The Domingo Rubio tidal system (Spain) is a prime example of highly polluted wetland where the polluter-pays principle cannot be applied. In addition to diffuse contamination from agriculture and water pollution caused by multiple anthropogenic sources (Barba-Brioso et al., 2010a), local soil contamination occurs by improper disposal of sulfide-rich wastes coming from past mining activities. As a result, the wetland soil adversely affected by acid and heavy metal contamination has become sterile and unproductive. Under these conditions, only some races, ecotypes or edaphoendemisms are able to colonize such altered soils. It is the case of *Erica andevalensis*, an endemic heather adapted to highly acidic soil conditions along the acidic soils of the Iberian Pyrite Belt (Márquez-García et al., 2006; Rossini-Oliva et al., 2018).

Notwithstanding the above, an incipient vegetation usually arises spontaneously over time owing to the tendency for natural processes in soils to attenuate contaminants. Initially, the vegetative cover is composed of annual grasses that are able to colonize technogenic soils developed on mine wastes. These pioneer plants play an interesting role in preventing erosion, promoting soil dynamics, and maintaining other living beings of the trophic change since they are primary producers. Alien plant species may also colonize highly altered soil ecosystems, as they are more resilient and competitive than native species in degraded areas (Dana et al., 2005). Thus, a controversial issue arises when the exotic plants play a significant role in the remediation of contaminated soils, but also pose a threat to biodiversity of native ecosystems (Mendez and Maier, 2008). Interestingly, the species *Nicotiana glauca* and *Euphorbia segetalis* are found growing on the sulfidic waste material improperly disposed of in the Domingo Rubio wetland, displaying a patchy distribution, and hence, they have been selected as the focus of this study. It can be hypothesized, therefore, that both species could tolerate high levels of PTEs in surrounding soils.

The genus *Nicotiana* comprises around 67 species distributed in America, South Pacific, Australia and Southwest of Africa. Some of them contain alkaloids derived from nicotinic acid (nicotine). *Nicotiana glauca* R.C. Graham is a glabrous scrub or small tree (up to 6 m tall), natural from South America (Argentina, Paraguay, and Bolivia) and extensively naturalized in the Mediterranean region. It is a nitrophilous plant commonly found on slopes, embankments, roadsides, river terraces, and well adapted to growing in sandy or stony and disturbed soils (Dana et al., 2005). *N. glauca* was included in the Spanish Catalogue of Invasive Alien Species (Royal Decree 630/2013) until 2015 when a ruling by the Supreme Court of Spain forced its removal from the list and, since then, its use in natural landscapes is allowed. The reason for the exclusion was that it has a great potential as an energy crop, thus helping to tackle climate change. This species has the ability to survive in heavily polluted soils (Barazani et al., 2004), and genes associated with metal tolerance have been encoded (Shingu et al., 2005).

*Euphorbia segetalis* L. is an annual, biennial, or perennial herb (up to 80 cm tall), native to Mediterranean Europe, North of Africa, and Macaronesia. It is indifferent to the substrate type and occurs in a wide range of habitats, including ruderal sites, crop fields, stony pastures and rocky areas of coastal places. This species has been phytochemically characterized by its diterpene content (Jakupovic et al., 1998) and antiviral and antimicrobial activities of triterpenes isolated from *E. segetalis* have been assessed (Madureira et al., 2003). Some species of the genus *Euphorbia* (Euphorbiaceae) produce a large amount of latex that can cause severe irritation of the skin and eyes. The latex has been extensively investigated from pharmacognostic and phytochemical points of view, and many interesting antitumor molecules have been characterized (Sahai et al., 1981). To our knowledge, this is the first time that *E. segetalis* is being considered for its potential as phytoaccumulator of PTEs in a contaminated soil.

This paper was aimed at determining the environmental conditions, mineral components and degree of contamination of the technogenic soil where *N. glauca* and *E. segetalis* are growing, and the concentrations of PTEs in different organ tissues (roots, stems and leaves) in order to quantify the uptake of contaminants from soil by plant roots and their transfer and accumulation in the above-ground biomass of these spontaneous pioneer plants. The final goal is to understand the potential impact of this contamination and how it can affect the environment.

## Materials and methods

### Geoenvironmental setting

**Fig. 1:**
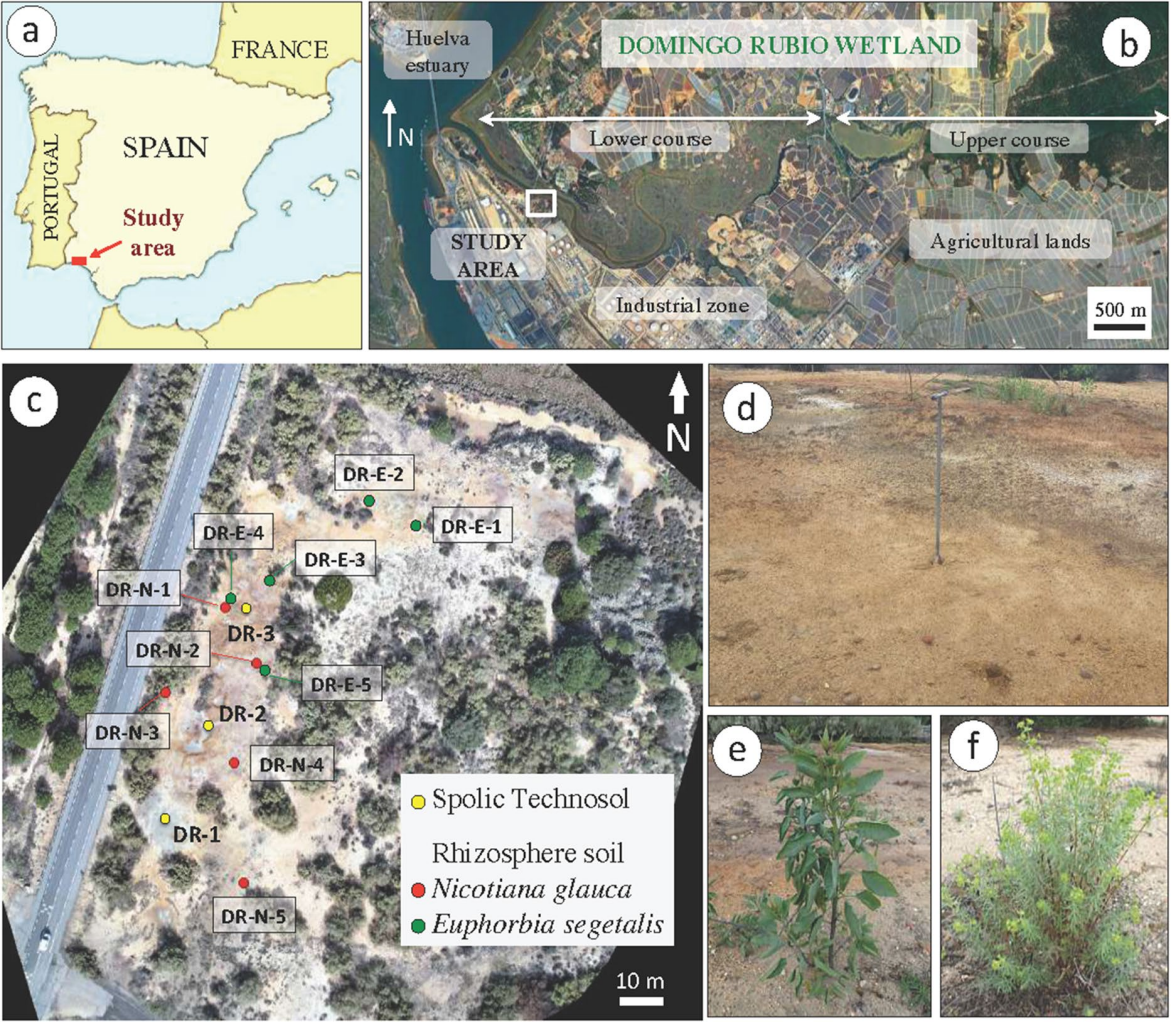
**a** Location map of the Domingo Rubio wetland area in the Huelva estuary (SW Spain). **b** Google Earth^®^ image showing the upper and lower course of the tidal channel, and the main activities developed around the study area. **c** Aerial view (drone image) of the sampling locations for soils and plants. **d** Soil sampling method using a hand auger. **e** *Nicotiana glauca* on site. **f** *Euphorbia segetalis* on site

The Domingo Rubio wetland is a small tidal channel 6 km in length connected to the confluence of the Tinto and Odiel rivers (Pendón et al., 1998), where they form the estuary of Huelva on the southwest coast of Spain (Fig. 1a, b). The tidal wetland is located in a flooded valley, which is incised into Plio-Pleistocene detritic deposits, occupying an area of about 480 ha. It has a mild Mediterranean climate moderated by the influence of the Atlantic Ocean (Csa in the Köppen–Geiger climate classification system), with average temperature of 9° C in winter and 27° C in summer, and with most of the rain (mean annual of 525 mm) falling in winter months.

The lower wetland area consists of a tidal marshland with a pronounced marine influence. The marsh soils (Salic Fluvisols according to World Reference Base for Soil Resources) are developed on fluvio-marine silty-clayey sediments, and the wetland vegetation is dominated by salt-tolerant plant species, such as *Spartina densiflora, Arthrocnemum macrostachyum* and *Halimione portulacoides* (Madejón et al., 2009). The upper wetland is a lacustrine area consisting of a freshwater reservoir artificially created by stream impoundment, which is inhabited by *Typha domingensis* and *Phragmites australis*, among others wetland species. This portion of the wetland is fed by Dehesa del Estero stream and many ephemeral creeks from a watershed impacted by intensive irrigated cropping operations (Barba-Brioso et al., 2010b).

The tideland area has been catalogued as Natural Area since 1989 and more recently as Special Protection Area of the Natura 2000 Network in October 2002 (Site code: ES6150003, Estero Domingo Rubio, Directive 92/43/EEC) hosting a total of 7 natural Habitats of Community Interest and 156 species (mainly birds) referred to in Article 4 of Directive, 2009/147/EC and listed in Annex II of Directive 92/43/EEC. Despite the legislative framework in place, the wetland receives high loads of pollutants (Online Resource 1) from diffuse agricultural and industrial sources (Barba-Brioso et al., 2009, 2010a) and acidic discharges from a Spolic Technosol developed on legacy sulfidic-rich materials due to improper past waste disposal practices.

### Plant and soil sampling

A stratified random soil sampling was undertaken in three representative sites of the Spolic Technosol developed on the abandoned sulfide-rich mine wastes (sites DR1, DR-2 and DR-3 in Fig. 1c). At each site, five topsoil samples (0–20 cm depth) were collected with an Edelman auger in crossing directions around the central sampling position (Fig. 1d) and bulked in the field to form one composite sample representing the site. The plants of *N. glauca* (Fig. 1e) and *E. segetalis* (Fig. 1f) and their associated soil samples were collected at ten sites in the wasteland area (five sites per plant species) where they spontaneously grew. Plants were harvested and separated into leaves, stems and roots. This sampling scheme allowed us to compare tissue metal concentrations in different groups of organs of the two only shrubs growing on the mine wastes. As a control, plant samples of the same species and soil samples from adjacent roots were collected from seemingly uncontaminated areas elsewhere. The soil from the rhizospheric zone was collected by vigorous hand-shaking and subsequent brushing of the root mass (Luster et al., 2009). Plant and soil samples were placed in zip-lock plastic bags and immediately transported to the laboratory for processing.

### Analytical methods

The soil samples were air dried, gently crushed to break the aggregates, passed through a 2-mm mesh sieve, and thoroughly mixed to ensure optimal homogenization. Soil reaction (pH), redox potential (Eh) and electrical conductivity (EC) were potentiometrically measured in a soil/deionized water suspension of 1:2.5 (w/v), after shaking for 15 min followed by a 30 min equilibration period. Aliquots of sieved material were ground in an agate mortar and pestle until getting a nearly uniform fine powder (< 63 μm) for mineralogical and chemical analyses.

Mineralogical identification of the major crystalline phases was carried out by powder X-ray diffraction (XRD) on a BRUKER AXS D8 Advance diffractometer, using CuKα radiation at 40 kV and 30 mA. Scans of randomly oriented bulk powders were run from 3 to 65° 2θ with a step size of 0.02° and a counting time of 0.6 s per step. Relative mineral abundance was estimated by empirical intensity factors weighting the integrated peak area values of diagnostic reflections (Kahle et al., 2002). Selected samples were examined by environmental scanning electron microscopy (ESEM) on a JEOL JSM-IT500 instrument coupled with energy-dispersive X-ray spectroscopy (EDS). Back-scattered electron (BSE) images and EDS spectra were acquired at 20 kV accelerating voltage to assist in the mineral identification of accessory heavy metal-bearing particles.

Soil samples were subjected to multi-acid (HF-HClO_4_-HNO_3_-HCl) digestion followed by inductively coupled plasma-optical emission spectroscopy (ICP-OES) analysis using an Agilent 735 ICP-OES instrument to determine the total concentrations of As, Cd, Co, Cr, Cu, Pb, and Zn. The analyses were performed at Activation Laboratories Ltd. (Ancaster, Ontario, Canada), which is accredited to the ISO/IEC 17025:2005 standard. Analytical quality control was monitored by using internationally certified reference materials (OREAS 13b, OREAS 98, OREAS 101b), blank samples and laboratory replicates to check the accuracy and precision of the data. The accuracy expressed as percent recovery ranged from 78.3 to 108.3%, and the precision expressed as percent relative standard deviation (RSD) was between 2.4% and 14.7%.

A one-step extraction test with a mild neutral salt solution was used to determine the phytoavailable concentrations of PTEs in the rhizosphere (Menzies et al., 2007). For this purpose, an aliquot (1 g) of each rhizosphere soil sample (< 2 mm) was subjected to extraction with 0.01 M CaCl_2_, in a soil/solution ratio of 1:10 (w/v), by end-over-end shaking at 30 rpm for 2 h (Houba et al., 1990; Rivera et al., 2016). The soil extract solution was then centrifuged at 3000 rpm for 10 min, and PTEs of concern were measured in the supernatant after filtration through 0.45 μm nylon filters. The analysis of PTEs in the digests was performed by ICP-MS on an Agilent 7700 instrument (Central Research Services, University of Huelva, Spain). The extraction procedure was repeated twice with similar results, and the mean value was taken. Extraction blanks were used to identify possible contamination.

In order to extract the PTEs of concern from plant tissues, all plant material (roots, stems and leaves) was washed thoroughly with tap water and rinsed with distilled water repeatedly to remove the surface dust particles and then, sonicated in a bath-type sonicator (Power Sonic 505) for 30 min. Afterward, the plant samples were dried in an oven at 40° C for 5 days, and ground to 0.5 mm in an IKA WERKE MF 10 grinder at 3250 rpm. The thicker stems were previously ground in a cutting mill RETSCH(R) model SM 2000 to pass through a 5 mm mesh sieve. An aliquot (0.05 g) of the ground sample was digested with 1 ml of concentrated HNO_3_ (Madejón et al., 2011) using a microwave oven at 350 W for 1.5 min. After cooling, 1.5 ml of H_2_O_2_ (30%) was added to the digest solution and the heating was repeated using the same microwave program. Finally, the resulting solution was diluted to a volume of 5 ml with Milli-Q water. The analysis of PTEs in the digests was performed with the Agilent 7700 ICP-MS. The same procedure was applied for control plant samples. The validity of the analytical procedure was assessed by analyzing a certified reference material of plant leaves of Oriental Basma Tobacco Leaves (INCT-OBTL-5) (Samczyński et al., 2012). The average precision was 7.8% RSD, and the average recovery was found to be 92.1%, which is indicative of good accuracy.

### Statistical treatment

All results are expressed as mean ± standard deviation. For statistical purposes, the results below the detection limit were assumed as half of the detection limit. Statistical differences between means were evaluated using Student’s t-test and analysis of variance (one-way ANOVA). Prior to ANOVA analysis, the homogeneity of variance was assessed by Levene’s test. The critical level of significance for all statistical tests was set at an α-value of 0.05 (95% confidence interval). Data statistical treatment was performed with the Statistica 10.0 and MS Excel 2016 software packages.

## Results and discussion

### Mineral composition and environmental soil conditions

The Spolic Technosol developed on sulfide-rich mine wastes left on the marshland differed drastically in mineral composition and physico-chemical properties from the natural soils in the wetland. It was essentially composed of pyrite, jarosite, gypsum, and quartz in highly variable proportions, with minor feldspars, hematite, and accessory barite and anglesite (Table 1). In dry periods, as surface soil dries out it appears covered with yellow efflorescences of readily soluble sulfate minerals, notably ferricopiapite, as reported by Barba-Brioso et al. (2010c). The secondary mineral assemblage was clearly a result of sulfurization processes driven by weathering of the pyrite-rich wastes, with sulfate minerals being the most abundant oxidation products. Consistently, the Technosol was extremely acid (pH values below 3.0) and strongly oxidant (Eh values ranging between + 659 and + 756 mV). These surface conditions are conducive to increased release of acidity, sulfate ions and PTEs, thus limiting the soil capability to support vegetation.

**Table 1:** Electrochemical parameters and mineral composition of the technogenic soil derived from abandoned mine wastes, of the soil surrounding the plant roots and their control soils

Sample		pH	Eh	EC	Mineral composition (wt %)						
		(in H_2_O)	(mV)	(mS cm^−1^)	Pyrite	Jarosite	Gypsum	Quartz	Clay minerals	Calcite	Minor phases (< 10%)
Technosol	DR-1	1.9	659	11.9	30 − 35	25 − 30		15 − 20			Ang, Brt, Fsp, Gp, Hem
	DR-2	2.2	756	5.6		15 − 20	70 − 80				Hem, Qz
	DR-3	2.7	697	3.3		35 − 40	45 − 50	30 − 35			Hem, Brt
Rhizosphere *N. glauca*	DR-N-1	7.4	415	2.65				15 − 20	45 − 50	10 − 15	Fsp, Gp, Jrs
	DR-N-2	7.8	393	0.47				40 − 45	40 − 45		Cal, Gp, Fsp
	DR-N-3	7.0	458	0.59				35 − 40	50 − 55		Gp, Fsp, Cal
	DR-N-4	7.7	398	0.13				60 − 65	25 − 30		Cal, Gp, Fsp
	DR-N-5	7.7	413	0.07				65 − 70	20 − 25		Gp, Fsp, Cal
Control	DR-N–C	7.5	415	0.68				40 − 45	35 − 40		Cal, Dol, Gp, Fsp
Rhizosphere *E. segetalis*	DR-E-1	7.1	403	1.77			10 − 15	45 − 50	25 − 30		Cal, Fsp
	DR-E-2	7.6	377	0.16				55 − 60	30 − 35		Gp, Fsp, Cal
	DR-E-3	7.9	384	0.64				35 − 40	45 − 50		Cal, Gp, Fsp
	DR-E-4	7.0	398	2.42				15 − 20	50 − 55	10 − 15	Fsp, Gp, Jrs
	DR-E-5	8.1	399	0.13				55 − 60	25 − 30		Fsp, Gp, Cal
Control	DR-E-C	7.0	453	0.12				60 − 65	25 − 30		Fsp, Gp, Cal

Mineral symbols: Ang (anglesite); Brt (barite); Cal (calcite); Dol (dolomite); Fsp (feldspars); Gp (gypsum); Hem (hematite); Jrs (jarosite); Qz (quartz)

*EC* electrical conductivity

Interestingly, the mineral constituents of the soil in which the plants have directly grown differed distinctly from those of the Technosol derived from the sulfidic wastes. The rhizosphere soil samples were composed mainly of quartz and clay minerals, together with minor amounts of feldspars and calcite, which coexisted with accessory gypsum and jarosite in some samples. It was noteworthy the occurrence of carbonates in most samples, indicating a good buffering capacity to neutralize the acid that has been produced, during the oxidative dissolution of pyrite in the abandoned wastes. In fact, soil active reaction was slightly alkaline, with pH_H2O_ values ranging from 7.0 to 8.1 (Table 1), which allowed specifically the growth of some species such as *N. glauca* and *E. segetalis*. The soil surrounding the plant roots showed Eh values (from + 377 to + 458 mV) and electrical conductivity values (up to a maximum of 2.65 mS cm^−1^) lower than those of the Technosol, and within the typical range reported for natural soils of the wetland (Barba-Brioso et al., 2021). The results suggested, therefore, that these species were able to grow in soil patches subjected to contrasting environmental conditions, having less adverse effects in terms of plant tolerance. An alternative hypothesis is the potential transformation of the rhizosphere by these plants using any kind of exudates to detoxify undesirable metal pollutants in soils (Chen et al., 2017). Further research is needed to understand the origin of this extreme modification of the rhizosphere and the reason why only these species are able to survive in this extremely hostile environment.

### Total and phytoavailable concentrations of trace elements

Total PTE concentrations in the technogenic soil greatly varied depending on the element considered (Table 2). The pyrite-rich material (sample DR-1) showed the highest concentrations of Pb (> 5000 mg kg^−1^), Cu (1710 mg kg^−1^), Zn (1610 mg kg^−1^), Co (51 mg kg^−1^), and Cd (8 mg kg^−1^), whereas the maximum concentration of As (1360 mg kg^−1^) and Cr (89 mg kg^−1^) were measured in the samples DR-2 and DR-3, respectively. In comparison with geochemical baseline concentrations of the local wetland soils (Barba-Brioso, 2011), the Technosol was found contaminated with all PTEs, except Cr. Specifically, the concentrations measured in the Technosol exceeded the local baseline concentrations up to 9.7 times for Pb, 8.9 for As, 8.0 for Cd, 3.4 for Co, 3.2 for Zn, and 2.3 for Cu. The Cr concentration fell within the normal range of the local soils since it is not regarded as a mine-source metal in the Iberian Pyrite Belt (Fernández-Caliani et al., 2009). It was also noticeable that the total concentrations of Pb, As and Cu exceeded the generic reference levels (GRL) or threshold limits established by current regulation (Junta de Andalucía, 2015) for agricultural and other land uses, including natural areas, indicating that exposure to these contaminants may result in an unacceptable risk level for human health or ecosystems. If the GRL are exceeded, the Spanish regulation on contaminated soils establishes that a site-specific risk assessment must be performed (Tarazona et al., 2005).

**Table 2:** Total trace element concentration in the technogenic soil derived from abandoned mine wastes

Trace element (mg kg^−1^)	As	Cd	Co	Cr	Cu	Pb	Zn
Detection Limit (DL)	3	0.3	1	1	1	3	1
Sample DR-1	1250	8.0	51	15	1710	> 5000	1610
Sample DR-2	1360	4.5	24	14	1570	4390	637
Sample DR-3	761	2.2	14	31	1160	2190	525
Baseline concentration*	153	< DL	15	89	732	515	508
Regulatory threshold**	36	25	24	10,000	595	275	10,000

Geochemical baseline concentrations of the local wetland soils and the generic reference levels established for soils of the Andalusia region (South Spain) are given for comparison

*Mean value in local wetland soils (Barba-Brioso, 2011)

**Junta de Andalucía (2015)

The rhizosphere soil of both plant species (Table 3) contained elevated concentrations of PTEs of concern, particularly Cu (up to 6370 mg kg^−1^) and Zn (up to 5250 mg kg^−1^) but also Pb (up to 2210 mg kg^−1^), As (up to 613 mg kg^−1^), and Cd (up to 18.7 mg kg^−1^), compared to local baseline concentrations, yielding contamination factors as high as 8.7 for Cu (sample DR-E-3), 10.3 for Zn (sample DR-E-4), 4.3 for Pb (sample DR-E-4), 4.0 for As (sample DR-N-1), and 18.7 for Cd (sample DR-N-1). Consistently, the PTE concentrations in rhizosphere were significantly above the control soil levels (p < 0.007) in all sampling sites, except for Cu in sample DR-N-5. This may be due to the fact that the control soil had an abnormally high concentration (1990 mg kg^−1^) arising from unexpected contamination in spite of their apparently natural trait. It could be possible due to diffuse contamination originated by re-suspension of waste material. It was also found that the sample DR-N-4 had an anomalously high content of Cr (223 mg kg^−1^) whose source was unclear. The pool of wastes from different sources/time slices made difficult the traceability of the materials. No significant differences were observed among the total PTE concentrations in the rhizosphere soil of the two studied plant species except for Cu, which was significantly higher in soil samples around the roots of *E. segetalis* than in those of *N. glauca* (*p* = 0.018).

**Table 3:** Trace element concentrations in the rhizospheric soil of *Nicotiana glauca* and *Euphorbia segetalis*

Trace element (mg kg^−1^)	As	Cd	Co	Cr	Cu	Pb	Zn
Detection Limit (DL)	3	0.3	1	1	1	3	1
*Rhizosphere N. glauca*							
DR-N-1	613	10.5	42	32	2640	1070	2150
DR-N-2	163	7.6	59	54	3210	814	4550
DR-N-3	86	1.1	7	30	1340	285	412
DR-N-4	54	3.2	20	223	3360	2210	1420
DR-N-5	69	1.7	6	17	1390	350	585
Mean	197	4.8	26.8	71.2	2388	946	1823
Std. deviation	236	4.1	23	86	972	778	1675
DR-N-control	11	< DL	4	41	65	34	98
*Rhizosphere E. segetalis*							
DR-E-1	208	2.5	20	28	2690	303	1220
DR-E-2	160	3.7	15	34	5730	408	1270
DR-E-3	135	6.0	19	37	6370	516	1540
DR-E-4	344	18.7	79	37	3460	1050	5250
DR-E-5	63	2.2	12	45	1110	339	1000
Mean	182	6.6	29	36	3872	523	2056
Std. deviation	105	6.9	28	6	2173	305	1796
DR-E-control	28	0.8	4	24	1990	107	273

Surprisingly, the levels of Cu, Zn and Cd in most rhizosphere samples were even higher than those observed in the sulfidic-rich wasteland. The contrasting soil pH values between the two soils could be a plausible explanation for the observed difference in PTE accumulation. Soil reaction is a factor of paramount importance concerning metal speciation and mobility in soil systems (McBride, 1994). Thus, a substantial fraction of Cu, Zn and Cd released by sulfide oxidation, which are relatively soluble under the acidic conditions prevailing in the Technosol, was immobilized to a large extent in some patches of neutral soil where *N. glauca* and *E. segetalis* grew spontaneously. Chemical fixation might have occurred either by precipitation, adsorption, or any other mechanism involving soil constituents with pH-dependent charge that tend to deprotonate with increasing pH, thus leading to increased metal retention. However, the highest concentrations of As and Pb were found in the Spolic Technosol, in association with relatively insoluble sulfate minerals like jarosite and anglesite. The precipitation of these secondary minerals at low pH was an efficient mechanism for decreasing the mobility of As and Pb at the source of contamination and could be the reason why some races of the studied species were able to survive.

It is generally agreed that total concentration of PTEs in soil does not provide predictive insights on mobility and availability of contaminants in relation to plant uptake (i.e., phytoavailability). The PTE pool that may be available in the rhizosphere for plant uptake (Madejón et al., 2011; Menzies et al., 2007) was assessed by single extraction with CaCl_2_.

The results of the analysis (Table 4) showed that the extracted element concentrations varied from undetectable levels to values below 8 mg kg^−1^. The maximum concentrations of Zn (7.40 mg kg^−1^), As (0.07 mg kg^−1^), Cd (0.06 mg kg^−1^), and Co (0.06 mg kg^−1^) were extracted from the soil surrounding the roots of *N. glauca*, whereas the highest content of extractable Cu (2.53 mg kg^−1^) was noted in the rhizosphere of *E. segetalis* (sample DR-E-2). The Pb concentration was below the detection limit by ICP methods in all soil extracts. The t-test showed no significant differences between both rhizospheric soils for the extractable concentrations of As, Cd, Co and Cu (*p* > 0.057), while the extractable Zn concentration was significantly higher in the rhizosphere soil of *N. glauca* (*p* = 0.027).

**Table 4:** Trace element concentrations extracted with CaCl_2_ from the rhizospheric soil of *Nicotiana glauca* and *Euphorbia segetalis* (< DL, below detection limit)

Element (µg kg^−1^)	As	Cd	Co	Cu	Pb	Zn
Detection Limit (DL)	3	1	5	47	7	29
*Rhizosphere N. glauca*						
DR-N-1	33 ± 2	6 ± 0.4	< DL	75 ± 6	< DL	< DL
DR-N-2	15 ± 0.3	< DL	< DL	< DL	< DL	< DL
DR-N-3	7.9 ± 0.2	46 ± 1	63 ± 1	925 ± 6	< DL	4382 ± 14
DR-N-4	66 ± 0.1	3.9 ± 0.03	< DL	805 ± 6	< DL	828 ± 6
DR-N-5	69 ± 0.5	59 ± 0.1	11 ± 0.1	1844 ± 30	< DL	7402 ± 143
Mean	38 ± 28	23 ± 27	15 ± 25	733 ± 690	< DL	2522 ± 3031
DR-N-Control	< DL	< DL	< DL	< DL	< DL	< DL
*Rhizosphere E. segetalis*						
DR-E-1	33 ± 2	3.0 ± 0.2	< DL	876 ± 38	< DL	51 ± 12
DR-E-2	46 ± 0.003	18 ± 0.1	13 ± 0.01	2570 ± 24	< DL	2340 ± 19
DR-E-3	8.6 ± 0.1	5.1 ± 0.1	< DL	388 ± 3	< DL	< DL
DR-E-4	22 ± 1	16 ± 1	< DL	65 ± 6	< DL	277 ± 21
DR-E-5	27 ± 1	4.4 ± 0.2	< DL	203 ± 1	< DL	< DL
Mean	27 ± 13	9 ± 7	4 ± 5	820 ± 949	< DL	534 ± 941
DR-E-Control	36 ± 0.01	104 ± 0.4	72 ± 0.03	14,085 ± 143	12 ± 0.4	98,872 ± 1301

Overall, the extractability, expressed as the amount of PTE extracted with CaCl_2_ relative to its total concentration in the soil rhizosphere, was practically negligible. The extractable fraction of contaminants accounted for less than 1% with a few exceptions concerning Cd (up to 4.18%) and Zn (up to 1.27%). The low extractability of PTEs with CaCl_2_ suggests that only a trivial fraction of PTEs may be available for plant uptake. In this approach, the phytoavailable pool includes water-soluble and non-specifically adsorbed PTEs that are retained on soil particles, notably clay minerals, by relatively weak electrostatic interaction. Unexpectedly, the PTEs were extracted from the rhizosphere control soil of *E. segetalis* to a greater extent than from the contaminated soil. As noted previously, this could be due to unforeseen contamination with potentially mobile PTEs.

### Trace element concentrations in plant tissues

A detailed comparative analysis of PTEs concentrations in plant tissues (Table 5) reflected significant differences between plant organs and species (ANOVA, *p* < 10^–5^), with extreme values ranging from 0.034 mg kg^−1^ to 13.39 mg kg^−1^ for As, 0.123–13.16 mg kg^−1^ for Cd, 0.105–6.757 mg kg^−1^ for Co, 19.90–395.9 mg kg^−1^ for Cu, 0.621–41.83 mg kg^−1^ for Pb, and 60.51–336.9 mg kg^−1^ for Zn. Among the PTEs under investigation, only the concentrations of Cu in leaf tissue were higher than those compiled by Kabata-Pendias (2011) as excessive or toxic values, and so may pose a potential risk to the food chain. It has detected the presence of numerous rabbits in the study area. There are many rabbit latrines and clear evidence of root predation of the studied species with unpredictable consequences on the biota.

**Table 5:** Trace element concentrations (mg kg^−1^) in plant organs of *Nicotiana glauca* and *Euphorbia segetalis*

Plant species	Plant organ	Sample	As	Cd	Co	Cu	Pb	Zn
*N. glauca*	Root	DR-N-1	13.39 ± 0.14	1.907 ± 0.026	1.426 ± 0.018	149.2 ± 0.8	41.83 ± 0.28	149.3 ± 0.1
		DR-N-2	9.617 ± 0.162	1.279 ± 0.029	2.673 ± 0.048	234.8 ± 1.7	34.50 ± 0.47	281.8 ± 1.4
		DR-N-3	6.717 ± 0.091	1.397 ± 0.023	0.766 ± 0.014	313.8 ± 2.4	26.94 ± 0.27	142.1 ± 0.6
		DR-N-4	1.792 ± 0.030	0.452 ± 0.011	0.228 ± 0.007	67.54 ± 0.47	7.243 ± 0.115	135.8 ± 0.6
		DR-N-5	5.772 ± 0.072	0.577 ± 0.011	0.327 ± 0.007	132.9 ± 0.4	32.37 ± 0.29	117.8 ± 0.2
		Control	1.377 ± 0.034	0.270 ± 0.006	0.601 ± 0.018	30.95 ± 0.63	3.436 ± 0.093	53.85 ± 0.96
	Stem	DR-N-1	1.578 ± 0.023	3.940 ± 0.065	6.757 ± 0.088	39.15 ± 0.38	4.154 ± 0.063	250.5 ± 0.6
		DR-N-3	0.288 ± 0.008	0.977 ± 0.015	3.776 ± 0.050	21.76 ± 0.26	0.621 ± 0.035	107.4 ± 0.5
		DR-N-4	0.034 ± 0.005	0.577 ± 0.008	2.850 ± 0.038	19.90 ± 0.25	0.706 ± 0.037	60.51 ± 0.44
		DR-N-5	0.496 ± 0.013	1.973 ± 0.041	2.562 ± 0.048	52.45 ± 0.73	4.776 ± 0.094	134.6 ± 1.1
		Control	0.024 ± 0.004	0.170 ± 0.002	1.045 ± 0.019	7.976 ± 0.188	0.118 ± 0.032	43.30 ± 0.51
	Leaf	DR-N-1	2.139 ± 0.020	13.16 ± 0.18	0.184 ± 0.004	128.6 ± 0.1	11.46 ± 0.08	336.9 ± 1.1
		DR-N-2	0.855 ± 0.022	6.488 ± 0.182	0.406 ± 0.013	97.95 ± 1.43	6.228 ± 0.154	307.5 ± 3.6
		DR-N-3	1.147 ± 0.028	3.589 ± 0.087	0.338 ± 0.012	148.0 ± 2.3	9.333 ± 0.184	184.1 ± 2.0
		DR-N-4	1.567 ± 0.034	3.906 ± 0.098	0.220 ± 0.008	215.4 ± 2.3	9.428 ± 0.184	224.9 ± 2.0
		DR-N-5	0.946 ± 0.021	2.962 ± 0.073	0.105 ± 0.006	124.7 ± 1.3	6.335 ± 0.133	163.1 ± 1.4
		Control	0.258 ± 0.010	0.225 ± 0.009	0.020 ± 0.005	31.78 ± 0.64	0.411 ± 0.049	58.99 ± 1.15
*E. segetalis*	Root	DR-E-1	6.377 ± 0.117	2.004 ± 0.047	0.777 ± 0.017	395.9 ± 3.5	9.561 ± 0.166	167.5 ± 1.2
		DR-E-2	7.423 ± 0.090	0.825 ± 0.012	0.800 ± 0.013	394.4 ± 2.5	14.05 ± 0.14	120.1 ± 0.4
		DR-E-3	3.176 ± 0.012	1.019 ± 0.006	0.811 ± 0.005	254.8 ± 0.6	8.304 ± 0.017	94.73 ± 0.48
		DR-E-4	9.358 ± 0.051	2.761 ± 0.025	0.981 ± 0.008	284.1 ± 0.0	23.48 ± 0.05	163.8 ± 0.7
		DR-E-5	7.485 ± 0.043	1.636 ± 0.015	1.145 ± 0.010	322.0 ± 0.0	39.12 ± 0.07	219.8 ± 1.1
		Control	1.519 ± 0.005	0.286 ± 0.000	0.313 ± 0.003	68.27 ± 0.21	3.920 ± 0.013	95.79 ± 0.68
	Stem	DR-E-1	1.509 ± 0.029	0.963 ± 0.019	0.761 ± 0.017	172.6 ± 1.9	6.040 ± 0.105	99.23 ± 0.86
		DR-E-2	2.614 ± 0.015	0.726 ± 0.005	0.981 ± 0.008	234.0 ± 0.1	9.239 ± 0.032	116.2 ± 0.5
		DR-E-3	1.786 ± 0.010	0.315 ± 0.001	0.451 ± 0.005	134.7 ± 0.2	4.968 ± 0.024	70.97 ± 0.21
		DR-E-4	4.425 ± 0.046	1.035 ± 0.013	0.270 ± 0.007	140.9 ± 0.7	14.42 ± 0.11	85.77 ± 0.23
		DR-E-5	0.831 ± 0.007	0.432 ± 0.002	0.390 ± 0.005	87.12 ± 0.01	4.563 ± 0.028	87.71 ± 0.28
		Control	0.558 ± 0.016	0.200 ± 0.004	1.349 ± 0.031	54.52 ± 0.89	2.652 ± 0.076	158.4 ± 1.6
	Leaf	DR-E-1	1.481 ± 0.027	0.618 ± 0.015	2.771 ± 0.050	154.1 ± 1.2	8.771 ± 0.145	118.7 ± 0.8
		DR-E-2	1.964 ± 0.022	0.656 ± 0.011	3.581 ± 0.040	121.2 ± 0.2	16.16 ± 0.13	162.4 ± 0.1
		DR-E-3	0.994 ± 0.009	0.123 ± 0.003	1.254 ± 0.012	51.73 ± 0.04	4.531 ± 0.043	88.94 ± 0.24
		DR-E-4	3.476 ± 0.051	0.766 ± 0.016	0.876 ± 0.015	87.22 ± 0.48	18.92 ± 0.22	116.2 ± 0.4
		DR-E-5	0.810 ± 0.001	0.195 ± 0.001	1.410 ± 0.002	54.16 ± 0.46	4.818 ± 0.004	103.5 ± 1.1
		Control	0.704 ± 0.004	0.138 ± 0.002	11.65 ± 0.05	60.49 ± 0.28	3.689 ± 0.026	380.5 ± 3.0

**Fig. 2:**
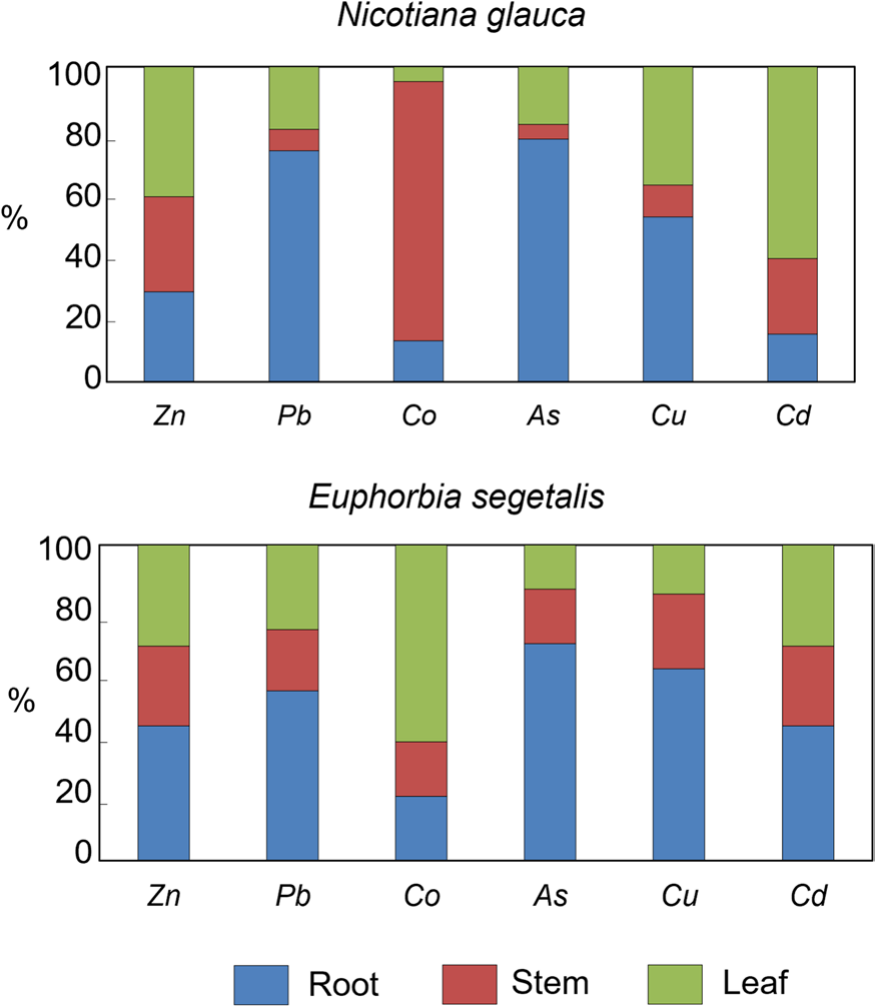
Partitioning of trace elements among the plant organs of *Nicotiana glauca* and* Euphorbia segetalis*

By studying distribution of PTEs among roots, stems and leaves, a similar pattern can be drawn from both species for all PTEs except Co (Fig. 2), although the fractionation pattern was somewhat more homogeneous in *E. segetalis*. Results from chemical partitioning in plant tissues showed that the roots of the two investigated species accumulate significantly more As, Cu and Pb than the above-ground biomass (*p* = 0.0001). The target organs of such PTEs accumulation appeared to be: root > leaf > stem in *N. glauca*, and root > stem > leaf in *E. segetalis*. By contrast, the mean fractions of Zn and Cd showed a significant decrease in roots relative to aerial parts of *N. glauc*a, but they did not exhibit any clear distribution pattern among the organs of *E. segetalis*. Unlike all aforementioned PTEs, Co appears to be preferentially partitioned both in stems of *N. glauca* and leaves of *E. segetalis*.

**Fig. 3:**
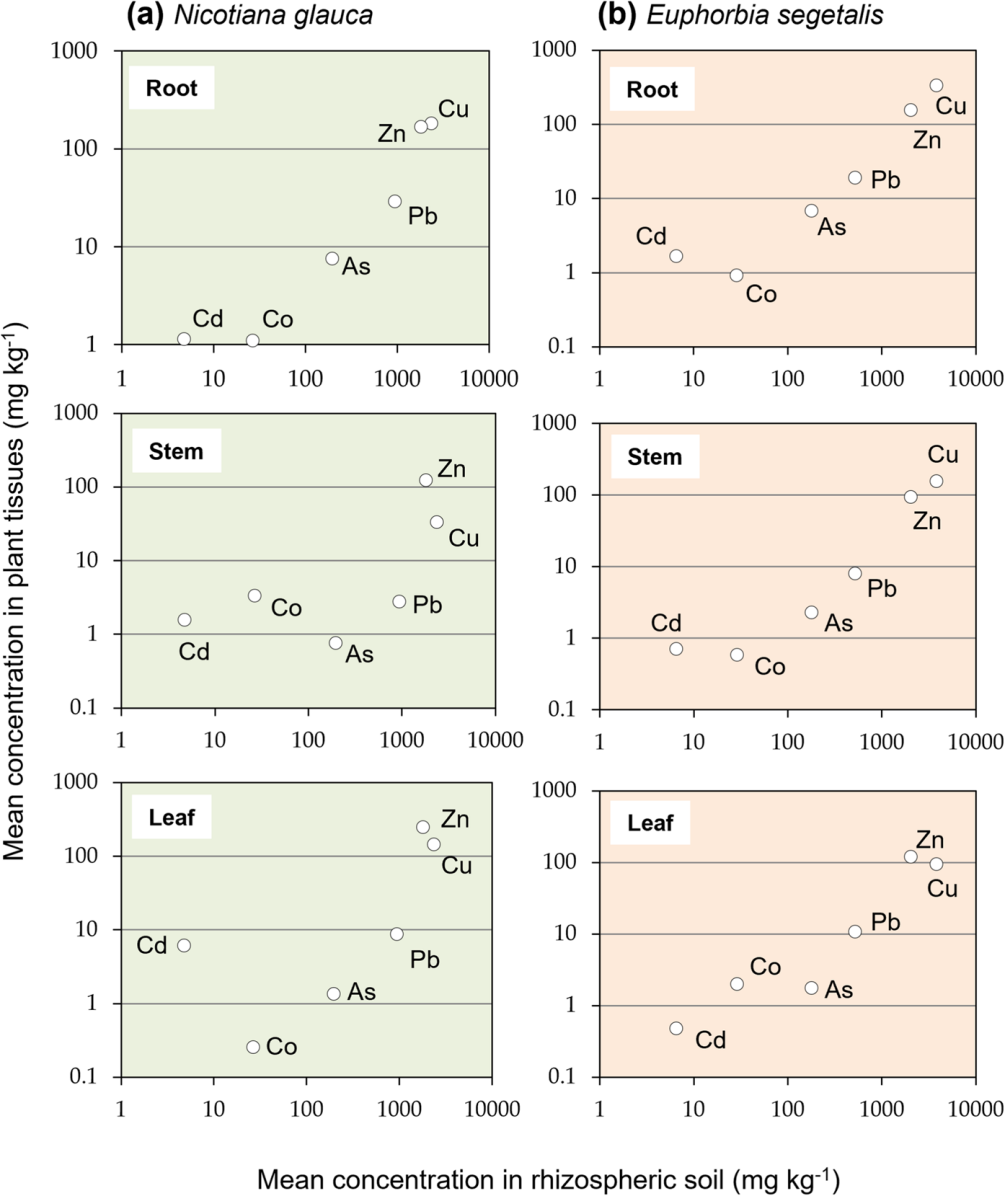
**Trace** elements mean concentration in organs vs mean concentration in rhizosphere soil of **a** *Nicotiana glauca* and **b** *Euphorbia segetalis*

**Fig. 4:**
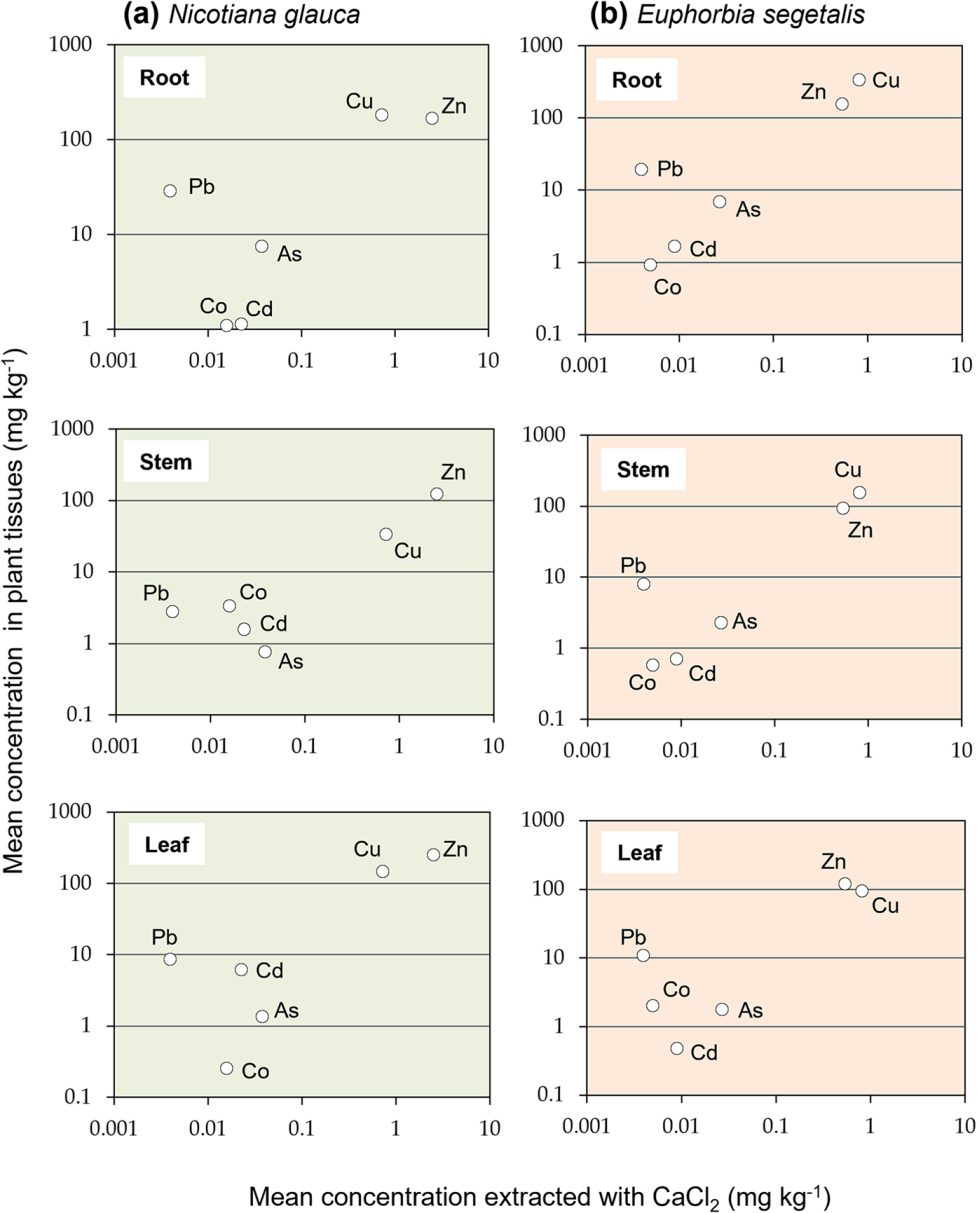
Trace elements mean concentration in organs vs CaCl_2_-extracted mean concentration in rhizosphere soil of **a** *Nicotiana glauca* and **b** *Euphorbia segetalis*

The total concentrations of PTEs measured in the narrow zone of soil subject to the influence of roots may be used as a primary indicator to assess the uptake and transmission of contaminants through the soil–plant system of *N. glauca* and *E. segetalis*. Biogeochemical processes in the rhizosphere zone are known to produce various organic compounds, which are effective in releasing PTEs from firmly fixed species in soil (Kabata-Pendias, 2011). Figure 3 depicts the relationship between mean concentrations of PTEs in rhizospheric soil and those in plant organs of the two species. In general, the distribution of PTEs appeared to follow a pattern that involves an increase in the mean concentration in both soil and plant tissues, with Cu and Zn being particularly implicated. It should be also noted the high level of Cd measured in the leaves of *N. glauca* compared to its content in soil immediately adjacent to roots. When the mean concentrations of PTEs in plant tissues are plotted versus the mean concentration of PTEs extracted with a standardized solution of CaCl_2_ (Fig. 4), which is considered that may be available in the rhizosphere for plant uptake, a similar picture emerged for *N. glauca* and *E. segetalis*. The highest CaCl_2_-extractable PTEs (i.e., Cu and Zn) were accumulated in plant organs to a greater extent than the others, although Pb showed a relatively high concentration in all organs of both plant species despite its low extraction yields. This could be explained by the fact that, under certain circumstances, the root exudates were capable of forming complexes with trace element ions in solution (Mench & Martin, 1991), thus enhancing Pb mobilization and uptake by plants.

The potential mobility of PTEs from soil to plants was evaluated by the soil-to-plant transfer coefficient (TC), which is a mobility index that records the average concentration of the whole plant (C_plant_), or certain plant parts, over that in rhizospheric soil (C_soil_) of each element of concern (Adriano, 2001; Antoniadis et al., 2017; Kumar et al., 2013; Shaheen & Rinklebe, 2015), as follows:

$${\text{TC }} = {\text{ C}}_{{{\text{plant}}}} /{\text{ C}}_{{{\text{soil}}}}$$

thus allowing feasible comparisons among PTEs, as well as among different plant tissues. TC has been used as indicator of bioavailability (Adriano, 2001) and to estimate the phytoremediation potential of plants (Yoon et al., 2006).

The soil–plant transfer coefficient values of PTEs in all parts of both plants were well below unity with the only exception of Cd in leaves of *N. glauca* (Table 6), indicating that a limited fraction of PTEs was actually solubilized by root exudates and transferred to plants despite the high degree of soil contamination. This finding suggests that roots acted as a barrier limiting the uptake of PTEs by plants. Interestingly, *N. glauca* can absorb Cd in considerable proportions from rhizosphere and accumulate it in its leaves under the same soil conditions. This is in line with other studies, showing that Cd has great potential to escape the soil–plant barrier (Chaney et al., 1999).

**Table 6:** Soil–plant transfer factors of trace elements for each plant organ of *Nicotiana glauca* and *Euphorbia segetalis*

Trace element	As	Cd	Co	Cu	Pb	Zn
*Nicotiana glauca*						
Root	0.038	0.234	0.040	0.075	0.030	0.091
Stem	0.004	0.322	0.123	0.014	0.003	0.067
Leaf	0.007	1.254	0.009	0.060	0.009	0.133
*Euphorbia segetalis*						
Root	0.037	0.250	0.031	0.085	0.036	0.075
Stem	0.012	0.105	0.020	0.040	0.015	0.045
Leaf	0.010	0.071	0.068	0.024	0.020	0.057

A better way to quantify the uptake of contaminants from soil by plant roots and their transfer and accumulation in above-ground biomass is to use the translocation factor (Baker, 1981), which can be estimated through the shoot–root quotient, as follows:

$${\text{TF }} = \, C_{{{\text{stem}}\;{\text{or}}\;{\text{leaves}}}} / \, C_{{{\text{root}}}}$$

where TF is the translocation factor, C_stem or leaves_ is the concentration of PTE in aboveground part of the plant (stem or leaves) and C_root_ is the concentration of PTE in root. A TF > 1 indicates that plants not only tolerate but utilize the PTEs in a beneficial way (Antoniadis et al., 2017).

**Fig. 5:**
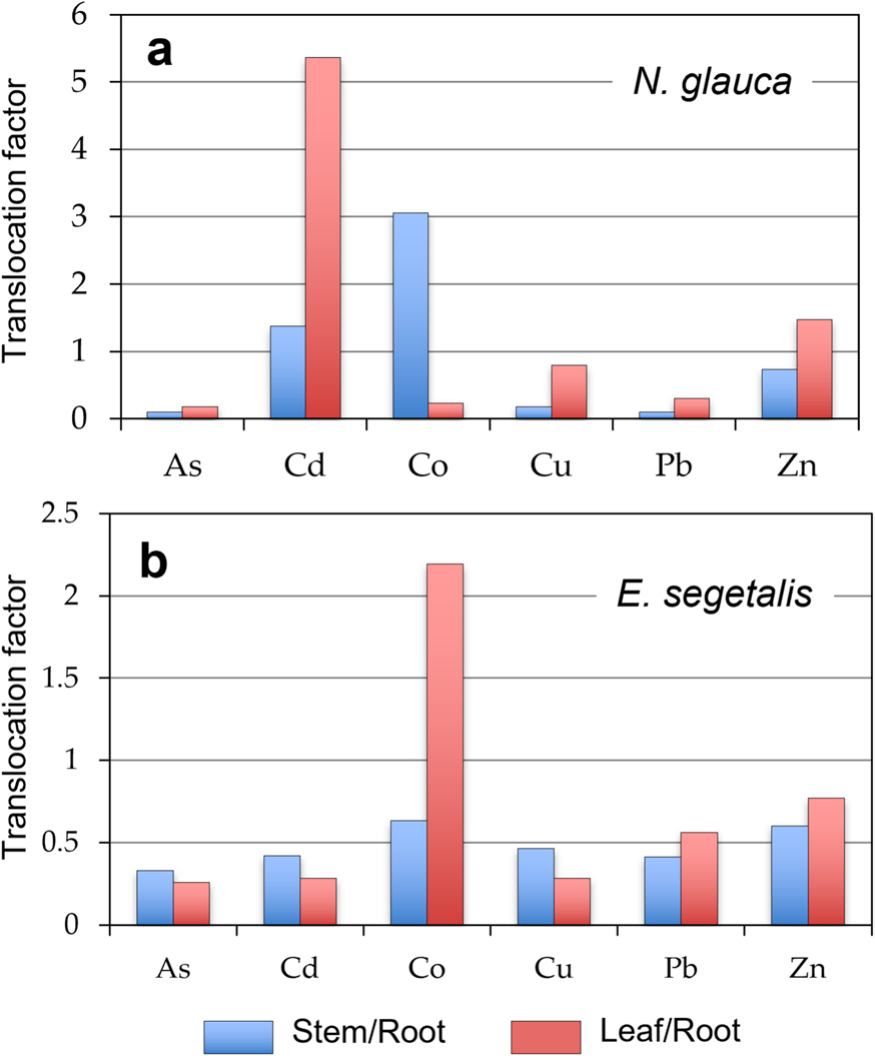
Translocation factors calculated as the ratio between trace element mean concentration in aerial parts (stems and leaves) and trace element mean concentrations in the roots of **a** *Nicotiana glauca* and **b** *Euphorbia segetalis*

As shown in Fig. 5, *N. glauca* translocated Cd effectively from the root to the stem (TF = 1.4) and, to a large extent, to the leaves (TF = 5.4), as well as Co from the root to the stem (TF = 3.1), and Zn from the root to the leaves (TF = 1.5), indicating that this wild plant species had high efficiency for phytoextraction of such elements from the contaminated soil. *N. Glauca* tolerance to PTEs has been previously reported (Barazani et al., 2004; Martínez-Fernández et al., 2014) related to the encode of a phytochelatin synthase and the production of nicotianamine synthetase that would promote some PTEs tolerance (Gajaje et al., 2021; Shingu et al., 2005). Instead, the capacity of *E. segetalis* was not effective in transferring PTEs from roots to shoots because the translocation factor values of PTEs were below unity except for Co, which appeared to be accumulated mainly in leaf tissues (TF = 2.2). This is a feature of metal excluder plant (Baker, 1981; Kumar et al., 2013). Therefore, marked differences in the PTE accumulating ability were found between both plant species.

## Conclusions

The soil developed on legacy mine wastes (Spolic Technosol) of the Natural Area *Estero Domingo Rubio* is highly acidic and strongly contaminated with sulfate and PTEs arising from oxidative dissolution of sulfidic material, which impose severe limitations for establishing vegetation. The results from this study led to the conclusion that, despite these unfavorable soil conditions for plant development and growth, *N. glauca* and *E. segetalis* are able to colonize heavily contaminated soil with a slightly alkaline reaction, occurring in isolated randomly distributed patches on bare land. The high acid-neutralizing capacity of the rhizospheric soil was effective in reducing PTEs mobilization, and consistently a limited fraction of phytoavailable PTEs (i.e., extractable with CaCl_2_) was transferred to plants. Soil acidity is regarded, therefore, as the key factor limiting plant development. Notwithstanding, the results of the plant tissue analysis showed that *N. glauca* and *E. segetalis* accumulated relatively high concentrations of As, Cu and Pb into their roots, reflecting the high degree of soil contamination, while the aerial parts of the plants were the target organs of Zn, Cd and Co. The establishment of these spontaneous metal-tolerant plants helps to mitigate the adverse impact of the mine wastes on the wetland, although the risk of PTEs entering the food chain needs to be evaluated. The new insights gained from this study provide criteria to assist in natural remediation in other legacy contaminated sites worldwide.

### Supplementary Information

Below is the link to the electronic supplementary material.

Landscape view (360 video) taken at 120 m by a DJI Air 2s drone from the study area. Close-up of Domingo Rubio tidal channel and its mouth into the Tinto river. In the background: Odiel-Tinto estuary and salt marshes, industrial, agricultural and urban areas, and oil refinery plant.
